# Short-Term Spinal Cord Stimulation or Pulsed Radiofrequency for Elderly Patients with Postherpetic Neuralgia: A Prospective Randomized Controlled Trial

**DOI:** 10.1155/2022/7055697

**Published:** 2022-04-27

**Authors:** Lei Sheng, Zihao Liu, Wang Zhou, Xiaojun Li, Xin Wang, Qingjuan Gong

**Affiliations:** ^1^Center for Rehabilitation Medicine, Department of Anesthesiology, Zhejiang Provincial People's Hospital (Affiliated People's Hospital, Hangzhou Medical College), Hangzhou, Zhejiang, China; ^2^Department of Anesthesiology, The First Affiliated Hospital of Guangzhou Medical University, Guangzhou, China; ^3^Department of Pain Management, The State Key Clinical Specialty in Pain Medicine, The Second Affiliated Hospital, Guangzhou Medical University, Guangzhou, China

## Abstract

**Background:**

Postherpetic neuralgia (PHN) is the most common and severe complication after varicella-zoster infection, especially in elderly patients. PHN is always refractory to treatment. Both pulsed radiofrequency (PRF) and short-term spinal cord stimulation (stSCS) have been used as effective analgesia methods in clinic. However, which technique could provide better pain relief remains unknown.

**Objectives:**

This study is aimed at evaluating the efficacy and safety of PRF and stSCS in elderly patients with PHN. *Study Design*. A prospective, randomized-controlled study. *Setting*. Department of Pain Management, the Second Affiliated Hospital of Guangzhou Medical University.

**Methods:**

A total of 70 elderly patients with PHN were equally randomized to the PRF group or stSCS group. Patients in the PRF group received PRF treatment, while patients in the stSCS group received stSCS treatment. The primary outcome was the effective rate. The secondary outcomes included the Visual Analogue Scale (VAS), the 36-Item Short Form Health Survey Questionnaire (SF-36), and the pregabalin dosage. All outcomes were evaluated at baseline and at different postoperative time points.

**Results:**

At 12 months after surgery, the effective rate reached 79.3% in stSCS group, while 42.1% in PRF group. The effective rate was significantly higher in the stSCS group than in the PRF group at 3, 6, and 12 months after surgery. VAS scores decreased significantly at each postoperative time point in both groups (*P* < 0.001). The VAS scores were significantly lower in the stSCS group than in the PRF group at 3, 6, and 12 months after surgery. SF-36 scores (bodily pain and the physical role) were significantly improved at each postoperative time point in both groups (*P* < 0.001). The SF-36 scores were significantly higher in the stSCS group than in the PRF group at some postoperative time points. The pregabalin dosage was significantly lower in the stSCS group than in the PRF group at 3, 6, and 12 months after surgery. *Limitations*. A single-center study with a relatively small sample size.

**Conclusions:**

Both PRF and stSCS are effective and safe neuromodulation techniques for elderly patients with PHN. However, stSCS could provide better and longer-lasting analgesic effect compared to PRF.

## 1. Introduction

Postherpetic neuralgia (PHN) is one of the most severe complications after infection of herpes zoster (HZ) [1]. The typical symptom of PHN is neuropathic pain distributed over the dermatomal innervation of the affected nerve for more than 3 months [2, 3]. It is estimated that more than 15% of the population worldwide will experience HZ infection in their lifetime, and the incidence will be significantly increasing among people aged over 50 years [4, 5]. Among these elderly HZ patients, complications will occur in almost half of them, especially PHN which has a high occurrence rate of 12.5% [2, 6]. After decades of research on PHZ, its exact neuropathological mechanisms are still not well understood [3]. Currently, effective treatments for PHZ mainly include oral analgesics, pulsed radiofrequency (PRF), and spinal cord stimulation (SCS) [1, 7–9]. For elderly patients with intractable PHN, oral drugs always fail to achieve complete pain relief and produce side effects as the drugs dose increases. Besides, the clinical efficacy of drugs is uncertain due to individual differences [1]. Hence, invasive therapies are always required for patients who are unresponsive to oral drugs.

PRF is commonly used as a neuromodulation technique in the field of chronic pain therapy [10]. PRF uses short pulsed current to create a high-voltage electric field around the target nerve, which can cause transient edema of the target nerve and further interfere with pain transmission. The effectiveness of PRF in treatment of PHN has been reported in lots of clinical studies [11–13]. SCS has been proven to be an effective interventional technique for patients with pain, especially for those with chronic and intractable neuropathic pain [8, 14]. By implanting electrodes in the epidural space of the appropriate spinal segment and further stimulating it, patients can feel paresthesia in specific area, which can reduce or cover their pain. Since introduced in 1967, SCS has been applied for chronic pain treatment in various diseases [15, 16]. In recent clinical studies, short-term spinal cord stimulation (stSCS) was used in PHZ patients, which produced definite therapeutic effect [17, 18]. Currently, both PRF and stSCS are clinically used for PHZ treatment, yet relevant clinical studies on the comparison between these two techniques are less, especially in elderly patient with intractable pain.

In the present study, we designed a prospective, randomized controlled trial to verify which therapy method is better for PHN patients aged over 50 years old.

## 2. Methods

### 2.1. Patients and Study Design

This study was a prospective, randomized, parallel group and controlled trial. It was conducted from January 1, 2015, to January 1, 2018, at the Department of Pain Management, the Second Affiliated Hospital of Guangzhou Medical University, Guangdong, China. This trial was carried out in accordance with the Declaration of Helsinki and approved by the Ethics Committee of the Second Affiliated Hospital of Guangzhou Medical University. All patients were informed the potential risks and complications of the trial and signed the written informed consent before therapy.

### 2.2. Inclusion and Exclusion Criteria

The inclusion criteria were as follows: (1) patients who were diagnosed with PHN according to the clinical diagnostic criteria [5]; (2) age ≥ 50 years old; (3) typical symptoms of PHN less than one year, such as pricking pain, burning pain, paresthesia, and pruritus; (4) the spinal nerves that were involved, including cervical, thoracic, and lumbar nerves; (5) Visual Analog Scale (VAS) score ≥ 4 points; and (6) pain refractory to conventional pharmacological (such as opiate analgesics, tricyclic antidepressants, anticonvulsants, and topical analgesics) or physical (such as percutaneous electrical nerve stimulation and acupuncture) therapies.

The exclusion criteria were as follows: (1) severe organ dysfunction, including brain, heart, lung, kidney, and liver diseases; (2) severe coagulation disorder or recent use of anticoagulant drugs; and (3) patients who had intellectual problems and were unable to complete self-evaluations, including VAS and 36-Item Short Form Health Survey Questionnaire (SF-36) [19].

### 2.3. Grouping and Sample Size

**Figure 1:**
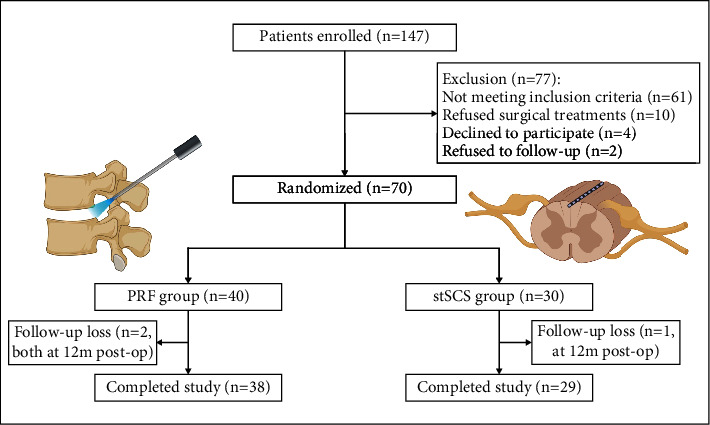
Study flowchart. Seventy patients were randomly assigned to the PRF group and stSCS group. PRF: pulsed radiofrequency; stSCS: short-term spinal cord stimulation.

One hundred and forty-seven patients with PHN were recruited in this study. Among them, 77 patients were excluded (61 patients did not meet the inclusion criteria; 10 patients refused surgical therapies; 4 patients declined to participate; and 2 patients refused to follow-up), and the remaining 70 patients were randomly assigned to the PRF group (*n* = 40) or the stSCS group (*n* = 30) by using the sealed envelope method (each patient randomly chooses one of the two envelopes containing the PRF group and the stSCS group, respectively) (Figure 1).

According to our pilot study, the effective rates of PRF and stSCS were 45% and 86%, respectively. We then estimated that the sample number was at least 25 in each group, which provided a power of 80% and a level of statistical significance of 0.05 (*α* = 0.05). Considering a potential dropout rate of 5%, we enrolled at least 27 patients in each group. In this study, the blinding method was as follows. All surgical procedures were performed by the same surgeon (Dr. Gong). Pain and function assessments at baseline and at each time point of follow-up were performed by the same investigator who did not know which group the subjects belonged to. The PRF/stSCS instrument was operated by a same nurse who did not participate in any treatment or follow-up.

### 2.4. Surgical Procedures (PRF and stSCS)

The procedures for PRF were as follows. The patient was placed supine (cervical nerve affected) or prone (thoracic and lumbar nerves affected) on the computer tomography (CT) treatment bed. Life signs (blood pressure, heart rate, and oxygen saturation) were continuously monitored. The target intervertebral foramen and the puncture route on the affected side were determined by CT scanning. After satisfactory local anesthesia, radiofrequency needle (20-G, length 100 mm for cervical/thoracic segment and 150 mm for lumbar segment) was inserted according to the predetermined path. Under CT guidance and sensation monitoring (50 Hz, 0.5 V; the radiofrequency instrument, R2000B, Beijing Neo Science Co., Ltd), the needle was slowly advanced until its tip reached the upper edge of the target intervertebral foramen. After withdrawal without blood or cerebrospinal fluid, 2 ml omnipaque contrast medium was injected to confirm the accurate position (the route of the spinal nerve can be visualized). Then, the therapeutic stimulation was performed at the following parameters: temperature, 42°C; frequency, 2 Hz; pulse width, 20 ms; duration, 600 s; and voltage, 40-100 V. The voltage was adjusted gradually from small (40 V) to large (100 V) until the patient felt sensation discomfort. The criterion for parameter adjustment was that the pain area was effectively covered by the electrical-induced numbness. The high-voltage, long-duration stimulation was performed twice in each patient, and the duration between two stimulations was 10 minutes.

The procedures for stSCS were as follows. The patient was placed prone on the digital subtraction angiography (DSA) treatment bed. Life signs were routinely monitored. The spinous process was located under fluoroscopy. The target therapeutic segment was determined according preoperative pain dermatome. After local anesthesia, a Tuohy needle was inserted into the epidural space under fluoroscopic guidance, followed by the implantation of a 1 × 8-contact stimulation electrode (Model: Medtronic 3861, Medtronic, Inc.). The stimulation lead was placed 1-2 mm lateral to the central spinous process (toward the affected side), with its tip adjusted to an appropriate anatomical level. The optimal therapeutic lead position was defined as a pleasant paresthesia coverage of more than 50% of the pain area. Each patient in the stSCS group received only one stimulation lead. After satisfactory positioning, we anchored the lead to the supraspinous ligament and connected it to the pulse generator (Model: Medtronic 3625, Medtronic, Inc.) through an extension cable. Then, a therapeutic short-term electrical stimulation was performed for 2 weeks at the following parameters: voltage, 1-3 V; frequency, 20-80 Hz; and pulse width, 210-450 *μ*s. During the treatment period, patients can control the stimulation level appropriately according to their own response to the paresthesia.

### 2.5. Pharmacologic Therapies

Before surgical treatments, all patients received a single oral medicine (pregabalin) for pain relief according to their pain severity. The preoperative dose of pregabalin was recorded as the baseline dose. After surgery, all patients were still administered pregabalin for pain management. The pregabalin dose was adjusted according to pain severity. During the clinical trial, all patients avoided other PHN-related pharmacologic therapies.

### 2.6. Primary Outcome

The primary outcome was the effective rate from baseline to the end of day 360. The effective rate is defined as the proportion of patients with at least a 50% reduction in VAS scores from baseline. The primary outcome was assessed at baseline (before surgery) and at days 1, 7, 30, 90, 180, and 360 after surgery.

### 2.7. Secondary Outcomes

The secondary outcomes included VAS, SF-36, and pregabalin dosage. The VAS is used to assess pain severity on a scale from 0 (no pain) to 10 (intolerable pain), with higher scores indicating more severe pain. The SF-36 is designed to assess the health status of patients from 8 dimensions. Each dimension was scored from 0 to 100, with higher scores indicating better health. In this study, we assessed the bodily pain and the physical role, the two dimensions most associated with pain. Patients took pregabalin two or three times a day according to their pain severity. The average pregabalin dosage (mg/d) was recorded in both groups. The secondary outcomes were assessed at baseline (before surgery) and at days 30, 90, 180, and 360 after surgery.

### 2.8. Adverse Events

All adverse events were recorded throughout follow-up, including hematoma at the puncture site, infection, pneumothorax, spinal cord injury, peripheral nerve injury, cerebrospinal fluid leakage, and electrode displacement.

### 2.9. Statistical Analysis

All statistical analyses were performed using SPSS version 22 (IBM Corp., Armonk, NY). Continuous variables and discrete variables were presented as the mean ± standard deviation and frequency, respectively. For continuous variables, the Shapiro-Wilk test was used to evaluate data normality. An independent-samples t-test or Wilcoxon rank-sum test were used for comparison between groups. One-way repeated measures ANOVA followed by the Bonferroni post hoc test was used to compare baseline and postoperative outcomes in each group. For discrete variables, Pearson's chi-square, chi-square continuity correction, or Fisher's exact test was used for comparison between groups. A *P* value < 0.05 was considered statistically significant.

## 3. Results

### 3.1. Patient Demographics

A total of 147 patients with HZ-related pain were enrolled initially, and 77 patients were excluded. Two patients in the PRF group were dropped out at 12 months after surgery, and one patient in the stSCS group was dropped out at 12 months after surgery (Figure 1). Data of these three patients were eliminated. Hence, the final number of patients for analysis was 38 in the PRF group and 29 in the stSCS group (Figure 1). The demographic information included age, gender, duration of PHN, involved area, and preoperative pregabalin dosage. No significant differences in the above characteristics were found between two groups (*P* > 0.05) (Table 1).

### 3.2. Primary Outcome

**Figure 2:**
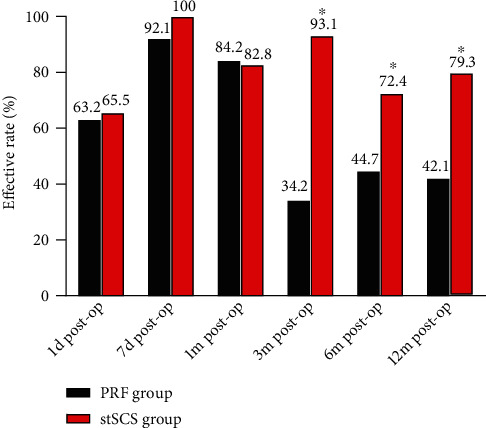
Postoperative effective rate. ^∗^*P* < 0.05 indicates the PRF group *vs.* stSCS group.

The effective rate was significantly higher in the stSCS group compared to the PRF group at months 3, 6, and 12 after surgery (*P* < 0.001, *P* = 0.023, and *P* = 0.002, respectively) (Figure 2). In the PRF group, the effective rate was 44.7% at 6 months after surgery and 42.1% at 12 months after surgery (Figure 2). However, in the stSCS group, the effective rate was 72.4% at 6 months after surgery and 79.3% at 12 months after surgery (Figure 2).

### 3.3. Secondary Outcomes

**Figure 3:**
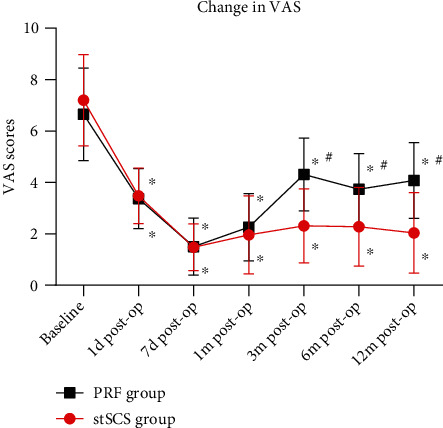
Pre- and postoperative VAS scores. ^∗^*P* < 0.001 indicates post-operation *vs.* baseline. ^#^*P* < 0.05 indicates PRF group *vs.* stSCS group.

**Figure 4:**
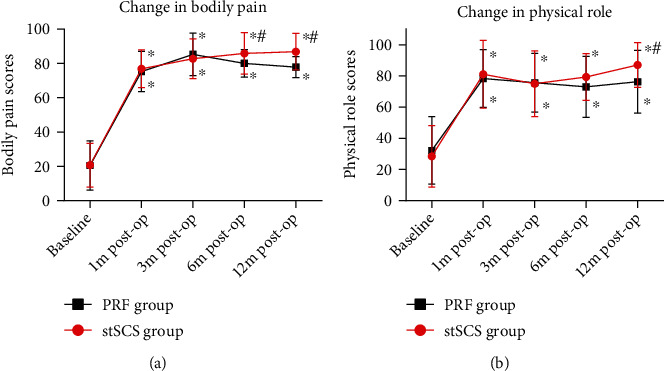
Pre- and postoperative SF-36 scores (bodily pain scores and physical role scores). ^∗^*P* < 0.001 indicates postoperation *vs.* baseline. ^#^*P* < 0.05 indicates PRF group *vs.* stSCS group.

**Figure 5:**
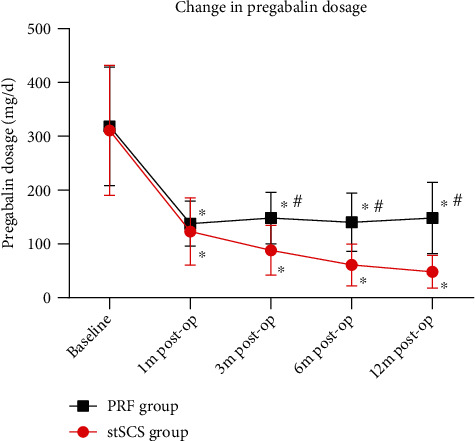
Pre- and postoperative pregabalin dosage. ^∗^*P* < 0.001 indicates postoperation *vs.* baseline. ^#^*P* < 0.05 indicates PRF group *vs.* stSCS group.

The average VAS scores before surgery were 318.42 ± 110.08 and 311.21 ± 120.93 in the PRF group and the stSCS group, respectively. No significant difference in preoperative VAS scores was found between two groups (*P* = 0.219) (Table 1). After surgery, the VAS scores significantly decreased in both groups at each time point, showing an obvious improvement of pain (*P* < 0.001) (Figure 3). However, the VAS scores were significantly lower in the stSCS group compared to the PRF group at months 3, 6, and 12 after surgery (*P* < 0.001) (Figure 3). No significant differences in preoperative bodily pain scores and physical role scores were found between two groups (*P* = 0.962 and *P* = 0.464, respectively) (Figure 4). After surgery, the scores significantly increased in both groups at each time point (*P* < 0.001) (Figure 4). In terms of bodily pain, the scores were significantly higher in the stSCS group compared to the PRF group at months 6 and 12 after surgery (*P* = 0.029 and *P* < 0.001, respectively) (Figure 4(a)). In terms of physical role, the scores were significantly higher in the stSCS group compared to the PRF group at month 12 after surgery (*P* = 0.017) (Figure 4(b)). After surgery, the pregabalin dosage significantly decreased in both groups at each time point (*P* < 0.001) (Figure 5). However, the dosage was significantly lower in the stSCS group compared to the PRF group at months 3, 6, and 12 after surgery (*P* < 0.001) (Figure 5).

### 3.4. Adverse Events

Slight hematoma at the puncture site occurred in 2 patients in the PRF group, which gradually subsided within 3 days after the surgery. No patient had infection, pneumothorax, spinal cord injury, peripheral nerve injury, cerebrospinal fluid leakage, electrode displacement, or other serious adverse events after surgical treatments.

## 4. Discussion

In this prospective randomized controlled study, both PRF and stSCS were effective interventional pain management techniques to relive pain and improve life quality in patients with PHN. The VAS scores were significantly reduced at all-time points of follow-up in both groups. However, the stSCS group showed lower VAS scores at 3, 6, and 12 months after surgery. The SF-36 scores significantly increased at all-time points of follow-up in both groups. Similarly, the stSCS group showed higher SF-36 scores at 12 months after surgery. In addition, the pregabalin dosage in the stSCS group was significantly lower than that in the PRF group at 3, 6, and 12 months after surgery. No serious adverse events occurred in both groups. These results suggested that stSCS provides better and durable pain relief than PRF in PHN patients.

PHN is the most severe complication of herpes zoster. After initial varicella-zoster virus infection, the viral particles invade nerve tissue and remain dormant in somatic sensory ganglia. When the body's cell-mediated immunity changes, the latent varicella-zoster virus can reactivate and replicate along the peripheral sensory nerves, causing neuronal damage and zoster-related pain in dermatomal distributions [5]. The dorsal root ganglion (DRG) is the enlarged tubercle of the dorsal root near each intervertebral foramen, where the cell bodies of first-order sensory afferent neurons are located. The main function of the DRG is to transmit the sensory impulses from peripheral nerve to the spinal cord and brain. DRG is considered to be a novel target for neuromodulation in the treatment of PHN. Here, we used PRF technique to precisely intervene in the function of DRG. PRF had been proven to be an effective technique for pain management [10–12, 20–22]. PRF technique generates a high-voltage but low-temperature (<40°C) environment around target nerve through high-frequency pulsed current, which can affect the conduction of pain sensory [23]. The underlying mechanism of PRF is attributed to various biological pathways in pain modulation, such as ion channels, neurotransmitters, synaptic function, and immune activity [24]. Since the first application in 1998, numerous studies have demonstrated that PRF on DRG can effectively relieve pain in patients with intractable pain, such as PHN [25]. In previous studies, pain relief started 2 or 3 days after PRF surgery and persisted for 2-6 months in the treatment of PHN [11, 12, 21, 26–28]. In our study, VAS scores decreased significantly at one day after operation. At 3, 6, and 12 months after surgery, the VAS scores showed a slight increase, but it was still significantly lower than baseline. Our results showed that PRF on DRG provided quick and lasting pain relief for PHN patients. In addition, the therapeutic electrical field generated by PRF is high-voltage but low-energy (low frequency and pulse width), which causes no or minimal damage to nerve tissue. Hence, patients did not experience uncomfortable symptoms of surgery-related neurological impairment after surgery. The quality of life also improved significantly after PRF surgery.

SCS is another representative technique of neuromodulation. To date, the exact analgesia mechanism of SCS is still unclear. It is considered that the ascending transmission of pain signals is reduced by electrical stimulation of the dorsal horn of spinal cord [29]. SCS-induced analgesia may also be attributed to the levels of neurotransmitters in the dorsal horn, which reduce zoster-related pain [30]. Since the first clinical application in 1967, SCS technique has developed rapidly [15]. In clinic, SCS is an ideal neuromodulation technique for the treatment of a variety of refractory or recurrent pain. Lots of previous studies have indicated that patients with neuropathic pain can benefit from SCS treatment [8, 17, 18, 21, 31–33]. Our results were consistent with the literature, showing significant pain relief after stSCS treatment. Moreover, the analgesia effect was maintained up to 1 year after operation, which had also been confirmed in some retrospective studies [18, 32]. In the present study, the effective rate was up to 72.4% at half year after surgery and 79.3% at one year after surgery. This result was consistent with a previous randomized controlled study which also showed a 70% effective rate [34]. In the present study, 6 patients in the stSCS group did not meet the criteria of effective rate in 12 months after operation. The preoperative duration of PHN in those six patients was 3.6, 5, 6, 6, 8, and 9.5 months. We considered that the failure to achieve an “effective” was partly due to the course of PHN. Yanamoto et al. reported that patients with a history of PHN less than 6 months could achieve better outcomes with temporary SCS treatment [32]. Hence, we suggested that patients with PHN should receive PRF or stSCS treatments as early as possible, if invasive treatments cannot be avoided [35].

Although postoperative VAS scores decreased significantly in both groups, the pain scores were lower in the stSCS group at 3, 6, and 12 months after treatment, indicating that stSCS therapy had better analgesic effect. This can also be reflected from the effective rate. The effective rate in the stSCS group was as high as 79.3% at one year after treatment, while only 42.1% in the PRF group. The possible reasons are as follows. First, the therapeutic mechanisms of the two techniques are different. The PRF technique is focused on DRG, collection of neuronal cell bodies of peripheral afferent sensory nerves. The stSCS technique is focused on the dorsal horn of spinal cord, the senior nerve center of the DRG. The effective therapeutic area of PRF is limited to the peripheral nerve, while stSCS could suppressed central sensitization. We considered that interventions in the higher-level central nervous system are superior to downstream interventions. Second, the stSCS treatment is a continuous microcurrent stimulation, while the PRF treatment is a temporary pulsed current stimulation. We believed that a 14-day long course of stSCS treatment is better than a 20-minute short course of PRF treatment. Third, the treatment area of PRF involves only one DRG, while the stimulation lead of stSCS has 8 stimulation sites. This indicated that the effective therapeutic dermatomes in the stSCS group were greater than those in the PRF group. In terms of effective rate, a certain number of patients in both groups failed to achieve the efficiency criteria. For patients in the PRF group, if the pain did not improve or recurred, a second PRF procedure or stSCS treatment was suggested [36]. For chronic or refractory PHN, implantation of permanent stimulation lead was recommended after efficacy testing by temporary electrode stimulation.

The main limitation of this trial is that it was a single-center study with a relatively small number of enrolled patients. A multicenter trial with large sample size is needed in the future. Nevertheless, our findings showed preliminary evidence that stSCS was superior to PRF in relieving pain in PHN patients.

## 5. Conclusions

Both PRF and stSCS could effectively relieve pain for patients with PHN. However, stSCS could provide better analgesic effect, lower pregabalin dosage, and better quality of life than PRF.

## Figures and Tables

**Table 1:** Preoperative characteristics of the patients.

Characteristics	PRF group (*n* = 38)	stSCS group (*n* = 29)	*P* value
Age (years)	68.29 ± 12.25	70.10 ± 10.24	0.522
Gender (male/female)	19/19	15/14	0.889
Duration of PHN (months)	3.19 ± 2.16	2.94 ± 2.33	0.657
Involved area			0.874
Cervical (%)	7 (18.4)	5 (17.2)	
Thoracic (%)	25 (65.8)	18 (62.1)	
Lumbar (%)	6 (15.8)	6 (20.7)	
VAS scores before surgery	6.66 ± 1.81	7.21 ± 1.78	0.219
Pregabalin dosage before surgery (mg/d)	318.42 ± 110.08	311.21 ± 120.93	0.800

PRF: pulsed radiofrequency; stSCS: short-term spinal cord stimulation; PHN: postherpetic neuralgia; VAS: visual analogue score.

## Data Availability

The data used to support the findings of present study are available from the corresponding author upon request.
